# Rehabilitation treatment of multiple sclerosis

**DOI:** 10.3389/fimmu.2023.1168821

**Published:** 2023-04-06

**Authors:** Haoyang Duan, Yuling Jing, Yinghua Li, Yawen Lian, Jianfang Li, Zhenlan Li

**Affiliations:** Department of Rehabilitation Medicine, First Hospital of jilin University, Changchun, China

**Keywords:** multiple sclerosis, pathogenesis and rehabilitation mechanism, assessment, new technique, progress

## Abstract

Multiple sclerosis is a slowly progressive disease, immunosuppressants and other drugs can delay the progression and progression of the disease, but the most patients will be left with varying degrees of neurological deficit symptoms, such as muscle weakness, muscle spasm, ataxia, sensory impairment, dysphagia, cognitive dysfunction, psychological disorders, etc. From the early stage of the disease to the stage of disease progression, professional rehabilitation treatment can reduce the functional dysfunction of multiple sclerosis patients, improve neurological function, and reduce family and social burdens. With the development of various new rehabilitation technologies such as transcranial magnetic stimulation, virtual reality technology, robot-assisted gait, telerehabilitation and transcranial direct current stimulation, the advantages of rehabilitation therapy in multiple sclerosis treatment have been further established, and more treatment means have also been provided for patients.

## 1 Introduction

Multiple sclerosis (MS) is an autoimmune inflammatory demyelinating disease, which is one of the leading causes of chronic neurological dysfunction (1). Nonstandard abbreviations and acronyms were shown in **Table 1**. According to statistics, there are about 2.5 million MS patients in the world, most of whom are 18-50 years old. The incidence rate of female patients is higher than that of male patients (2). In the clinical classification of MS, the most common type is the relapsing-remitting. Some patients may be converted to secondary progressive type, and 15% of patients have continuous progressive disability at the onset, which is the primary progressive type (3, 4). The classic MS is common in European and American populations, while most of them in China are relapsing optic neuromyelitis. MS is characterized by recurrent attacks, which can cause the continuous accumulation of nerve function defects, thus leaving a part of nerve dysfunction, such as muscle weakness, muscle spasm, tremor and ataxia, fatigue, sensory disorders, defecation disorders, sexual dysfunction, pain, dysarthria and dysphagia, cognitive dysfunction, mental and psychological disorders, resulting in gradually limited ability of daily life, It has seriously affected the health and social activities of patients (5, 6).

**Table 1:** Nonstandard Abbreviations and Acronyms.

Nonstandard Abbreviations and Acronyms	
MS	multiple sclerosis
BBB	blood brain barrier
CNS	central nervous system
OL	oligodendrocyte
DSS	disability status scale
EDSS	expanded disability status scale
MRDMS	minimal record of disability for multiple sclerosis
FS	functional systems
ADL	activities of daily living
IADL	instrumental activity of daily living
MSQOI	multiple sclerosis quality of life
SF-36	36-item short-form health survey
FSS	fatigue severity scale
6MWT	6-min walk test
ESS	environment status scale
SES	socioeconomic scale
MMSE	the mini-mental state examination
SEFCI	screening examination for cognitive impairment
AVLT	auditory verbal learning test
WMS	Wechsler memory scale
TMS	transcranial magnetic stimulation
rTMS	repetitive transcranial magnetic stimulation
PFC	prefrontal cortex
MC	motor cortex
VR	virtual reality
RAGT	robot-assisted gait training
PSQI	Pittsburgh sleep quality index
SIBT	sensory integration balance training
SIBT	sensory integration balance training
TUG	the timed up and go test
PHQ	the patient health questionnaire
MSIS-29	the MS impact scale-29
MSWS-12	the MS walking scale–12
BDI	Beck Depression Inventory

The treatment methods of MS include disease recurrence control, disease modification therapy and symptomatic treatment (7). Research has proved that cannabinoid drugs can relieve muscle spasms, tremors, pain and other symptoms caused by MS, and play a neuroprotective role by inhibiting neurotoxicity and regulating the immune system (8). So far, although immunosuppressants can delay the progression and deterioration of the disease, there is no effective drug treatment that can completely block the advance and cure the disease in MS patients (9). The latest research found that rehabilitation treatment can improve the functional status of patients and reduce their disability level, which is an important treatment method to improve the ability of daily living (10, 11). As an essential method of treating MS, rehabilitation therapy has become a hot spot in international medical research in recent years, and has also been paid more and more attention by developing countries. With the rapid development of transcranial magnetic stimulation(TMS), virtual reality(VR),robot-assisted gait training(RAGT), telerehabilitation (TR),transcranial direct current stimulation (tDCS) and other technologies, the means of rehabilitation treatment for MS patients are also more diversified and modern, further laying the status and advantages of rehabilitation medicine in MS treatment, and significantly improving the treatment effect of MS patients (12). This article reviews the progress of rehabilitation treatment for MS patients, aiming to provide reliable theoretical basis for the treatment of MS patients from the perspective of rehabilitation treatment,to promote the attention of countries around the world, especially developing countries, to the treatment of MS by rehabilitation medicine, and thus improve the treatment effect of MS patients.

## 2 Pathogenesis and rehabilitation mechanism of MS

MS basic pathological characteristics are focal demyelination, inflammatory reaction and gliosis, while axons are relatively preserved (13). At present, the specific pathogenesis is not clear. Paintlia et al. believed that some factors activated the peripheral immune system mediated by T cells and B cells, releasing a large number of inflammatory factors, attacking the blood-brain barrier (BBB) and infiltrating into the central nervous system (CNS) (14). Yeung et al. believed that changes in the inflammatory microenvironment of CNS led to the proliferation of glial cells and the secretion of inflammatory factors to attack foreign bodies, and the excessive secretion of inflammatory factors damaged oligodendrocyte (OL), leading to necrosis and apoptosis, followed by myelin shedding (15). Kasper et al. suggested that the immune system plays a major role in the pathogenesis of MS, the lesion involves white and gray matter in the spinal cord and CNS. The main pathological changes are the activation of T cells and B cells, secretion of various inflammatory factors, migration to the CNS, resulting in inflammatory infiltration of tissues, apoptosis and necrosis of OL, hyperplasia of MG and AST. Subsequently, demyelination occurs,eventually leading to neurodegeneration and loss of neuronal function (16, 17). Nishino et al. suggested that inflammatory factors and myelin fragments stimulated MG polarization to M1 type, and at the same time stimulated AST activation and secretion of inflammatory cytokines and chemokines, in addition, inflammatory factors secreted by MG and AST can aggravate demyelination and further aggravate the severity of MS (18). Sparaco et al. suggested that high levels of estrogen may be protective in women with MS because they found that the severity of the disease was significantly reduced in women with MS at the end of pregnancy (19).

Recent studies have shown that the pathogenesis of MS involves a variety of factors, which may be the result of the interaction of glial cells, immune factors,and environmental and nutritional factors (20, 21). We believe that the main mechanism may be that some predisposing factors act on patients with genetic susceptibility in a specific environment, inducing abnormal autoimmune response and causing MS. Immune mechanism is the central link of the pathogenesis of MS, including cellular immunity, humoral immunity and other aspects.

Rehabilitation therapy reduces inflammatory cytokines in the peripheral immune system, protects the central nervous system, slows neurodegeneration, induces neuroplasticity, and ultimately slows the progression of the disease (22, 23). The study by White et al. showed that after rehabilitation training, the levels of inflammatory mediators such as interleukin-4, interleukin-10, and C-reactive protein decreased in patients, which can reduce the inflammatory response of MS (24). Hotting et al. found that the levels of neurotrophic factors and growth factors were significantly higher in patients in the rehabilitation training group than in the control group (25). In addition, rehabilitation training can also relieve symptoms such as fatigue and weakness in MS patients by changing their pathophysiological processes (26).

## 3 Current status of MS rehabilitation

During the treatment of MS, medications have certain side effects and may have adverse effects on other functions (27). Specialized rehabilitation therapy can reduce the condition and dysfunction of MS patients, so rehabilitation therapy has received more and more attention.

### 3.1 MS rehabilitation assessment

Rehabilitation evaluation is the premise and basis of rehabilitation treatment. Through rehabilitation assessment, we can fully understand the dysfunction of MS patients, which can provide a basis for the formulation and revision of rehabilitation treatment plans.

MS has the characteristics of the multi-system disease, which leads to a variety of functional disorders in patients, such as limb movement disorders, sensory disorders, cognitive function changes, speech and swallowing disorders, bowel and stool dysfunction, ataxia, walking instability and other symptoms, which determine the complexity of rehabilitation evaluation content, therefore, the focus of evaluation is the dysfunction of function and living ability. The Disability Status Scale (DSS) was first developed by Kurtzke in 1955 on a 10-point scale, with 0 representing regular and 10 representing death, but the scale cannot distinguish small changes in disease severity (28). In the 1980s and 1990s, Kurtzke developed the Extended Disability Status Scale (EDSS) based on the DSS to assess MS dysfunction in detail (29). In 1985, the MS International Consortium established the minimal record of disability for MS (MRDMS). The disability section was developed according to the Kurtzke EDSS, which provides a detailed assessment of functional and personal disability in MS. The assessment is divided into two parts: 1) functional systems (FS), which is divided into eight systems and each system is divided into different grades; 2) The EDSS, which ranges from 0 to 10 points (30).

The activities of daily living scale (ADL), Operational Activities of Daily Living scale (IADL) and Multiple Sclerosis Quality of Life (MSQOL) were generally used to assess the living ability (31–33). The MSQLI, is a modular and specific assessment scale that not only includes the 36-item Short Form Health Survey (SF-36), but also includes nine specific clinical symptom assessments: fatigue, pain, bladder function, gastrointestinal function, mood, perception and cognitive function, visual function, sexual satisfaction, and social relationships (34). The fatigue severity Score (FSS) is commonly used to evaluate fatigue (35), and the 6-minute walk test (6MWT) is used to evaluate motor function (36). The Fatigue Scale-14 (FS-14) consists of 14 items, each of which is a fatigue-related problem, used to measure the severity of fatigue symptoms of MS and evaluate clinical efficacy (37).

Environmental status scale (ESS) and socioeconomic scale (SES) can be used to evaluate the social disability of MS (38, 39). Cognitive impairment can be assessed by comprehensive cognitive function screening scale and memory function assessment.

The comprehensive cognitive function screening scales included MS Screening Examination for Cognitive Impairment (SEFCI), Mini-mental State Examination (MMSE), etc. The Rey Auditory Verbal Learning Test (AVLT) and Wechsler Memory Scale (WMS-R) were used to assess memory function (40–43). Motor functions such as muscle strength, muscle tone, range of motion, limb coordination, balance and walking ability can also be evaluated. In addition, the patient’s sensory function, speech and swallowing function need to be evaluated.

### 3.2 Rehabilitation treatment of MS

MS presents complex and diverse dysfunction. The purpose of its rehabilitation treatment is to maintain and improve its function and improve the quality of life to the greatest extent. The principles of rehabilitation treatment should be targeted and vary from person to person. The content of rehabilitation treatment includes sports training, sensory training, psychological rehabilitation, cystorectal (second stool) function training, speech and swallowing training, cognitive function, rehabilitation education, etc. Specific measures include (but are not limited to): exercise therapy, occupational therapy, muscle strength exercise, application of assistive devices, physical factor therapy, percutaneous nerve stimulation, vibration therapy, acupuncture, psychological intervention, etc. (44, 45).

### 3.3 Family rehabilitation treatment of MS patient

The standard and planned systematic rehabilitation training for MS patients in the early stage can delay the occurrence of neurological dysfunction and reduce post-disease disability. The research of Asano et al. showed that the dysfunction of MS patients recovered the fastest in the first 3 months of illness, but the function of patients still improved after 12 months of illness (46). Therefore, it is suggested that our patients should receive comprehensive rehabilitation treatment and management for at least 12 months. Short-term rehabilitation treatment can help reduce the dysfunction of MS patients. However, a large number of MS patients lack long-term rehabilitation treatment, resulting in unsatisfactory rehabilitation effects (47). So we suggest that patients should continue to carry out family rehabilitation training after discharge.Professional rehabilitation doctors or rehabilitation therapists should systematically guide these rehabilitation training.

## 4 Application of new technology in MS rehabilitation treatment

In recent years, with the continuous deepening of relevant research on TMS, VR, RAGT, TR, tDCS and so on, many practical and innovative interventions have emerged in the rehabilitation technology of MS patients. These rehabilitation treatment technologies will open up a broader space for the treatment of MS and help to further improve the rehabilitation treatment effect of MS patients. Individual study characteristics, treatment characteristics, assessments, and outcomes (**Table 2**).

**Table 2:** Individual Study Characteristics, Treatment Characteristics, Assessments, and Outcomes.

Rehabilitation techniques	Study	Design	Stimulation Site	Parameter /Session	Assessments	Results
TMS	Korzhova et al., 2019 (48)	Divided randomly into three groups HF-rTMS (20 Hz)or iTBS or sham stimulation.	motor cortex(M1)	20Hz 80% MT 1200 pulses 2 weeks or bursts 5 Hz, 35 Hz, 80% MT 1200 pulses 2 weeks	MAS, SESS	Both HF-rTMS and iTBS can improve secondary spasticity in multiple sclerosis
	Gaede et al., 2017 (49)	Divided randomly into three groups(5Hz or 18Hz or sham stimulation)	PFC M1	5Hz 90% RMT 800 pulses 6 weeks or 18Hz 120%RMT 1800 pulses 6 weeks	FEE, Beck Depression Inventory scores.	The fatigue relief rate of PFC group and M1 group was higher than that of the false stimulation group;All patients with fatigue at BL showed significant improvement after treatment
	Hulst et al., 2017 (50)	MS group and healthy controls	DLPFC	10Hz 80% RMT 3000 pulses	Wechsler Adult, Intelligence Scale, Functional MRI	TMS therapy improves the accuracy and cognitive function of task oriented training in patients with MS
	Centonze et al., 2007 (51)	MS group	motor cortex(M1)	5Hz 100% RMT 1000 pulses 5 days	urodynamic evaluation	TMS can regulate detrusor activity and urination symptoms in multiple sclerosis, but has no effect on urinary storage function
VR	Munari et al 2020 (52)	RAGT + VR group and VR group	5 minutes for positioning the patient on the device, 30 minutes for RAGT, and 5minutes for removing the patient	40 minutes/day, 2 days/week total of 12 sessions.	Quality of Life-54, 2-MinutesWalk Test, 10-Meter WalkingTest, BBS	The BBS score of VR combined with robot training group was significantly higher than that of robot training group (P<0.05)
	Casuso-Holgado et al.2018 (53)	meta-analysis	PubMed PEDro CDSR CINHAL	All databases were searched from their inception until February 2018	PEDro scale	VR balance training is more effective than no intervention for postural control improvement
	Peruzzi et al 2016 (54)	Control group experimental group	treadmill training VR+treadmill training	45min/time,three times/week for six weeks total of 18 sessions	the six-minute walk test,Berg	VR can improve the walking endurance and speed, cadence and stride length, lower limb joint ranges of motion and powers, during single and dual task gait.
	Jonsdottir et al 2019 (55)	experimental group	a serious games	45min/time total of 12 sessions	the Nine Hole Peg Test, BBT	VR can improve upper limb motor function and help with family rehabilitation therapy
	Maggio et al 2022 (56)	Control group group+sVRT	evaluate the effect of sVRT	5 min warm-up+ 5 min strengthen+ 20 min gait + CR training,60min/time,3times/week, total of 24 sessions	MoCA,BDI,ROCF,ROCF COPY,ROCF IR,ROCF DR, MSQOL-54	VR cognitive training can potentiate MS patients’ rehabilitation outcome, with positive results on both motor and cognitive performance.
RAGT	Gandoli et al 2014 (57)	The RAGT group and The SIBT group	Compare RAGT with SIBT	50min/time,2 times/week, total of 12 sessions	walking speed,Breg	Within groups comparisons showed that both groups had improved balance, and only in RAGT did improvements in gait speed be found
	Dalgas et al 2011 (58)	A systematic review	none	Summary of other documents	6-min walking distance,20-meter walking velocity,Stride length,	RAGT training not only provides more effective support for walking training but also simulates a near-normal gait
	Straudi et al 2020 (59)	RAGT group or CT group	overground walking or RAGT	12 2-hour training sessions over a 4-week period.	6MWT,BBS,TUG,FSS, PHQ,SF-36, MSIS-29 MSWS-12	At the end of treatment with respect to baseline, both groups significantly improved gait speed (p<0.001)
TR	Charvet et al 2017 (60)	experimental group	active control of ordinary computer games	15 minutes gaming,Each daily training session consisted four exercises chosen from an active set of six,	Neuropsychological Test	Home-based TR training can improve the cognitive function of MS patients
	Jeong et al 2021 (61)	Control group experimental group	exercise program on a daily life	home-based individualized exercise+TR system	MSQOL-54	TR has a positive effect on the symptoms and quality of life of MS
	Jeong et al 2020 (62)	experimental group	TR system on the quality of sleep	use the system for 3 months	PSQI	TR system can improve the sleep quality of MS patients
tDCS	Mori et al 2010 (63)	False stimulation and tDCS group	the posterior fossa	Eligible studies of PD,ET and PSP	VAS Quality of life	tDCS can improve the pain and quality of life and can reduce the chronic central pain of MS patients, and the action time can achieve an ideal effect.
	Workman et al 2020 (64)	False stimulation and tDCS group	M1	2 mA 20min/time, total of 5 days	FSS,VAS,BDI	the knee extensor fatigue, fatigue and pain of patients in the tDCS group decreased, but the depression score did not change significantly

### 4.1 Transcranial magnetic stimulation

TMS causes axon depolarization increases the excitability of the corticospinal system, stimulates the plasticity of neurons, enhances the ability of synaptic transmission, and improves the responsiveness of the nervous system, to achieve the goal of targeted treatment (65). rTMS has good temporal and spatial resolution, good tolerance and almost no side effects (66).

Korzhova et al. evaluated the effect of TMS or intermittent Theta explosive magnetic stimulation (iTBS) on patients with MS secondary spasms. At the end of treatment, the modified Ashworth scale (MAS) scores of patients in both TMS and iTBS groups were significantly improved, but there was no significant improvement in the sham stimulation control group (48). Gaede et al. recruited 33 MS patients, who were randomly assigned to receive high-frequency stimulation of the prefrontal cortex (PFC), the primary motor cortex (M1) or false stimulation. After 6 weeks, a follow-up evaluation of fatigue severity scale (FSS) was conducted. The fatigue relief rate of the PFC group and M1 group was higher than that of the false stimulation group, and there was no obvious adverse reaction (49). Hulst studied the effect of TMS on the cognitive function of MS patients, and found that the MS patients treated with TMS showed activation of the frontal lobe, and the accuracy of task-oriented training was also improved, which improved the cognitive function of MS patients to a certain extent (50). Centonze et al. treated 10 MS patients with TMS, of which 6 patients had decreased detrusor activity, 3 patients had excessive detrusor activity, 9 patients had improved urination symptoms, but no improvement in urine storage function (51).

TMS is one of the most mature non-invasive brain stimulation technologies. It can directly regulate the central nervous system and has advantages that other treatment methods do not have. However, TMS stimulation parameters, including frequency, intensity, stimulation mode, location, treatment frequency, etc., need further multi-center and large sample studies to determine the optimal treatment plan.

### 4.2 Virtual reality technology

VR technology stimulates the brain through high-intensity, multi-sensory, repetitive, task-oriented strong feedback, and intervenes in motor, cognitive and sensory functions, so that patients can immerse in the virtual environment and achieve the ideal effect of rehabilitation training. Rehabilitation training through VR technology can promote the activation of the mirror neuron system of MS patients, resulting in cortical and subcortical changes in the brain, and further stimulate the synaptic reorganization and remyelination of the brain’s motor regions.Some studies have found that rehabilitation training through VR technology can stimulate synaptic reorganization and remyelination in brain motor regions,and rehabilitation training carried out under VR helps improve the balance, movement and cognitive function of MS patients (67, 68).

MS can cause extensive dysfunction. Rehabilitation training is carried out under VR technology to improve the balance, movement and cognitive function of MS patients. Munari et al. divided MS patients into VR combined with robot training group and robot training group. After 6 weeks of rehabilitation training, the BBS score of VR combined with robot training group was significantly higher than that of robot training group (P<0.05) (52). However, Casuso-Holgado et al. reviewed the VR balance function rehabilitation training of MS patients and included five studies using BBS as a measuring tool. The results showed that there was no statistically significant difference in the balance function improvement effect between the VR group and the conventional training group (P>0.05) (53).

Peruzzi et al. designed a single-blind randomized controlled trial to observe the effect of VR training on the gait of MS patients. The subjects in the control group received treadmill training, and the subjects in the experimental group received treadmill training based on VR. The results showed that the walking endurance and speed, stride frequency and stride length, range of motion and strength of lower limb joints of the two groups of subjects were significantly improved, The improvement of the balance function in the experimental group was significantly higher than that in the control group (P<0.05) (54). Jonsdottir et al. applied VR technology to treat the upper limb motor dysfunction of MS patients, and found that VR technology can improve the upper limb motor function of patients with nervous system diseases. Importantly, it is helpful for rehabilitation treatment at home (55). Maggio evaluated the impact of semi-immersive VR training on cognitive and motor disorders of patients with MS, investigated cognitive and motor results through clinical and neuropsychological scales, and observed significant improvement in cognitive parameters and motor scores (56).

The application of VR technology in the field of rehabilitation has its own inherent advantages, but at present, there is little research on the application of VR in the rehabilitation training of MS patients in China. In the long run, the future development needs a unified consensus standard to standardize the intervention strategy.

### 4.3 Robot-assisted gait training

RAGT through a weight loss support system and exoskeleton support is able to generate sufficient repetitive gait movement, increase the training intensity and time, improve the gait characteristics of patients, help improve balance, increase the stability of lower limbs, and reduce the risk of falls (69). RAGT can improve lower extremity muscle strength but also reduce spasticity. In China, RAGT is relatively concentrated in hospitals or universities in provinces with developed economies and high scientific research levels.

Gandoli et al. suggested that lower extremity RAGT training can improve the gait, postural control, balance function, disability level, and activities of daily living in patients with MS, and the possible mechanism is that rehabilitation training on specific tasks may enhance neuroplasticity (57). Dalgas et al. found that RAGT training not only provides more effective support for walking training but also simulates a near-normal gait (58). In a study by Lamers et al., RAGT was used to treat more severe MS patients with better clinical outcomes than conventional treatment (70). Straudi et al. used a powered exoskeleton robot to treat MS patients, which increased the muscle strength of the patients, thus improving the transfer ability and activities of daily living of the patients (59).

RAGT training can provide MS patients with a training environment conducive to balance control and unlimited repetitions, enabling them to achieve early walking. Through weight-loss walking training, not only can improve the participation level of MS patients but also the spasticity of their lower limb muscle tissue can be reduced by continuous stretching (71). RAGT can provide different training modes according to patients’ specific conditions, including passive motion mode, active-assisted motion mode, resistance motion mode, and a bilateral mirror motion mode.

In China, the research on RAGT has significant differences among different regional institutions. In the future, it is necessary to strengthen the communication between developing countries and different countries or institutions abroad, and strengthen the degree of scientific research cooperation, to promote the application of RAGT in MS patients.

### 4.4 Telerehabilitation

Telemedicine was first proposed in the 1960s and developed rapidly in developed countries. TR as a rehabilitation model uses Internet communication technology to administer treatment between physicians and patients across time and space. Its convenient, fast, cheap, and time-free characteristics are of great significance to continuing patient rehabilitation training after discharge (72, 73).

Charvet et al. conducted a double-blind, randomized, controlled trial to recruit 135 patients with MS, of which 74 patients received home-based TR training, and the other 61 patients underwent home-based rehabilitation training by themselves for 12 weeks. The results showed that the cognitive function of MS patients receiving home-based TR training was significantly improved, indicating that TR training can better improve the cognitive function of MS patients (60). Jeong et al. observed the effect of rehabilitation training on the quality of life of MS patients. The MS patients in the observation group received rehabilitation training under the guidance of the TR system, while the MS patients in the control group did not receive the guidance of the TR system. The results showed that TR positively affects the symptoms and quality of life of MS (61). Jeong et al. also discussed the effect of the TR system on the sleep quality of MS patients, and found that there was a significant correlation between the exercise time spent by MS and sleep efficiency and quality, so it is necessary to carry out TR treatment (62).

In general, the TR system is a kind of home-based rehabilitation supplementary means with high acceptance and good safety for patients, which is beneficial to the rehabilitation of MS patients after discharge, and reduces the care burden of patients’ families, to truly achieve the whole-process rehabilitation of MS patients.

At present, developed countries have established relatively complete TR systems, while TR in most developing countries is still at the initial stage of exploration and research. Developing countries can learn from the experience of developed countries, improve TR technology and improve the rehabilitation guidance plan, to guide MS patients to carry out TR more comprehensively and effectively in the future.

### 4.5 Transcranial direct current stimulation

TDCS is a non-invasive nerve regulation technique. This technology transmits weak direct current to the surface of the cerebral cortex through at least two electrodes to achieve the purpose of neural regulation. Compared with other neural control methods, tDCS has certain portability, safety and ease of use (74).

In recent years, tDCS has been widely used as a tool to regulate cognitive function, improve mental disorders and neurological disorders. However, at present, the mechanism of tDCS on MS is not clear, and it is mainly used to alleviate the symptoms of MS.

Mori et al. divided MS patients into two groups in a randomized, double-blind experiment. One group received false stimulation and the other group received anode-tDCS for five consecutive days. The research results showed that the pain and quality of life of patients with anode-stimulation were significantly improved, and no depression and anxiety were found. This proves that anode tDCS can reduce the chronic central pain of MS patients, and the action time can achieve an ideal effect. Although the mechanism of anode tDCS in treating MS disease is still unclear, it is likely to be closely related to the remodeling of brain nerve function (63). Workman et al. evaluated the effect of tDCS on pain, fatigue and depression in MS patients. In this double-blind, pseudo-control, randomized study, moderately disabled women with MS were included in the study. The patients were randomly divided into tDCS stimulation or pseudo-tDCS stimulation. After 5 days of treatment, the knee extensor fatigue, fatigue and pain of patients in the tDCS group decreased, but the depression score did not change significantly (64).

The most common adverse reaction of tDCS is that during the stimulation, the scalp will be accompanied by slight tingling, itching and slight dizziness. These reactions will disappear within a few hours after the stimulation, without any long-term side effects, and have high safety.

## 5 Summary

MS patients have many clinical manifestations and related complications, and are prone to relapse. There are many clinical and rehabilitation issues that need further research, such as bladder rectal dysfunction, pain, and autonomic dysfunction. With the development of various new rehabilitation technologies, such as TMS technology, VR technology, RAGT, TR, tDCS, etc,making rehabilitation therapy become an indispensable treatment method for MS. However, in some developing countries, it is necessary to learn from the experience of developed countries and apply advanced rehabilitation treatment technologies to the treatment of MS patients. In the future, with the continuous deepening of relevant research, more advanced rehabilitation treatment methods will be applied to the treatment of MS.

## Author contributions

All authors contributed to the article and approved the submitted version.
